# Rapid purification of HU protein from *Halobacillus karajensis*

**Published:** 2014-03

**Authors:** Parinaz Ghadam, Rana Samadi

**Affiliations:** Department of Biology, Faculty of sciences, Alzahra University, Tehran, Iran

**Keywords:** DNA binding proteins, Histone-like protein, HBsu, HU, *Halobacillus karajensis*

## Abstract

The histone-like protein HU is the most-abundant DNA-binding protein in bacteria. The HU protein non-specifically binds and bends DNA as a hetero- or homodimer, and can participate in DNA supercoiling and DNA condensation. It also takes part in DNA functions such as replication, recombination, and repair. HU does not recognize any specific sequences but shows a certain degree of specificity to cruciform DNA and repair intermediates such as nick, gap, bulge, etc. To understand the features of HU binding to DNA and repair intermediates, a fast and easy HU protein purification method is required. Here we report a two-step purification procedure of HU from* Halobacillus karajensis* (the gram positive and moderately halophilic bacteria isolated from Karaj surface soil). The method of HU purification allows obtaining a pure non-tagged protein. Salting out and ion exchange chromatography were applied for purification, and the purified protein was identified by immunoblotting. Results showed that the molecular weight of the purified protein was approximately 11 kDa which is immunologically similar to the *Bacillus subtilis* HU protein (HBsu).

## INTRODUCTION

Bacteria possess a group of small, basic, plentiful and DNA-associated proteins which have several roles in the modification of chromosome topology, site specific recombination and DNA replication [1, 2]. These proteins are similar to eukaryotic histones in electrostatic charge, low molecular weight, sufficiently high cell quantities, and binding to DNA; they are thus known as histone-like proteins [3]. Histone-like proteins have specific structural and regulatory functions in bacteria [2]. One such protein is HU [4], which is described as an *Escherichia coli (E. coli)* strain U93 histone-like protein, found in almost all *Eubacteria* [5, 6]. It is a basic, heat-stable, small protein consisting of two monomers, HU-1 and HU-2 [7, 8].

HU prefers to bind to DNA containing structural aberration, such as single- or double-stranded breaks and replicative forks, but the bind is usually non-specific [9, 10]. The functional role of HU in bacteria is to participate in DNA supercoiling. In addition to the structural role, HU takes part in DNA-dependant activities such as the initiation of replication, transcription, and site-specific recombination [11].

The homolog of HU protein in *Bacillus subtilis* (*B. subtilis*) is HBsu which is encoded by the *hbs* gene [12]. HBsu has a high similarity with *E. coli* HU. It demonstrates 57% and 51% identical amino acids with the HU subunits HU-2 and HU-1 respectively. *B. subtilis* has one gene for encoding HBsu histone-like protein, and is therefore, a homodimer protein [13]. 

Among the different *Bacillus* species sequenced, conservation in the HU protein is more than 80% [14]. For example, 87% identity and 94% similarity has been reported to exist between the HBsu of the mesophilic *B. subtilis* and HBst, the HU protein of *Bacillus stearthermophilus* [15]. 

*Halobacillus karajensis (H. karajensis) *is a *Halobacillus* species isolated from Iran’s soil [16]. There is no information about the structure and function of the HU protein of *Halobacillus *genus. To determine the features of a protein like HU, it should be purified. In this study, a fast and easy HU purification procedure with a high yield was introduced, and the immunoblotting method was used to analyze the purified protein. The results showed that the molecular weight and immunological properties of HU protein from *H. karajensis* were similar to HBsu from *B. subtilis.*

## MATERIALS AND METHODS

**Bacterial strain and growth media:** The research was performed with *Halobacillus karajensis* strain Ma-2^T ^and *Bacillus subtilis* ATCC 6633t, obtained from Tehran University and the Iranian Research Organization for Science and Technology (IROST) respectively. *H. karajensis* was cultured in NB (10% NaCl) and NA (10% NaCl), pH 7-7.5 at 34°C*. B .subtilis *was cultured in TSA (Tryptone Soya Agar) and TSB (Tryptone Soya Broth).

**Preparation of protein extracts:** *H. karajensis* cells growing in NB (10% NaCl) were harvested in 500 ml culture medium 14 h after inoculation (180 rpm, 34^°^C) and suspended at four volumes of its wet weight in Buffer A (20 mM Tris-HCl (pH 7.8), 20 mM NH_4_Cl, 10 mM (CH_3_COO)_2_Mg, 5 mM 2-merecaptoethanol). The suspension was incubated with lysozyme (Nedayefanrah) (10 mg/ml) at 37^°^C for 1 h before sonication at 0^°^C. The sonicated suspension was centrifuged at 15000 rpm for 20 min and 45000 rpm for 3 h at 4^°^C. The protein extract of *Bacillus subtilis* was prepared according to the above procedures [17].

**Ammonium sulfate precipitation:** Cell extract was fractionated by ammonium sulfate solution (45-90%). After centrifugation at 28000 rpm for 20 min, precipitates were re-suspended in Buffer B (20 mM Tris-HCl, pH 7.8, Mg (CH3COO)_2 _10 mM, 2-mercaptoethanol 5 mM, and sucrose 0.25 M) and dialyzed against this buffer. The resultant solution was analyzed with SDS PAGE.

**Anion-Exchange Chromatography:** The resultant dialysate (500 µl) was loaded onto a DEAE-Sepharose CL-6B column (0.5 x 5 cm) pre-equilibrated with Buffer B. The unadsorbed proteins were washed from the column with the same buffer, and the rest was fractionated with a linear gradient of 0 to 2.0 M NaCl in buffer B with a flow rate of 75 ml/h. The eluted fractions were dialyzed against buffer B. All fractions were analyzed with SDS PAGE.

**Poly acryl amide gel electrophoresis**: A Laemmli SDS poly acrylamide gel (15% separating gel, pH 8.8 and 4% stacking gel, pH 6.8) was prepared. The enzyme preparations were diluted in a 5X sample buffer containing 0.125 M Tris-HCl, pH 6.8, glycerol, beta-mercaptoethanol and bromphenol blue as the tracking dye. The samples were then loaded into electrophoresis wells after boiling for 5 min. Electrophoresis was carried out at room temperature with 120 mV, until the tracking dye ran off the gel. The gel was stained with silver staining [19] or Coomassie Brilliant Blue R-250 in 50% methanol and 3.5% acetic acid for 30 min, followed by destaining in 10% methanol and 10 % acetic acid overnight or until the gel background was clear [18].

**Immunoblotting:** Proteins were electrophoretically separated by 15% SDS polyacrylamide gel transferred to a nitrocellulose membrane (Whatman protran BA83) in an electrode buffer (25mM Tris Base, 192mM glycine, 20% (v/v) Methanol, pH 8.3) by 200 mA for 2 h at room temperature. The paper with the immobilized protein bands was incubated for 1 h at 37^°^C with 1% bovine serum albumin in PBS and washed three times for 15 min with PBS. These and all successive incubation and washing steps were performed while solutions were gently shaken. The reaction with the diluted anti HBsu antiserum [20, 21] with 1% bovine serum albumin in PBS (1:1000 diluted) was carried out at 4^°^C overnight, followed by the removal of excess antibody by washing with PBS/Tween 20 [0.05% (v/v)] for three times in 15 min. The paper was incubated with diluted horseradish peroxidase conjugated goat anti-rabbit IgG (Sigma) with 1% bovine serum albumin in PBS (1:500 diluted) for 2 h at room temperature. Another washing was then carried out and the paper strip was incubated with the substrate solution for 15 min at 37^°^C (1.2 ml of 0.3% 4-chloro-1-naphtol in methanol was mixed with 20 ml PBS and 20 µl H_2_O_2_). The membrane was later washed with water for five minutes [20].

## RESULTS AND DISCUSSION

The roles of the HU protein in the regulation of transcription, initiation of replication, and repair of DNA [2] are important topics for research. However, no perfect investigations into the HU protein of the *Halobacillus *genus have been reported so far*.* The results of the present study on this protein in *H. karajensis* isolated from Iran’s soil [16] can thus be useful in widespread research.

**Figure 1:**
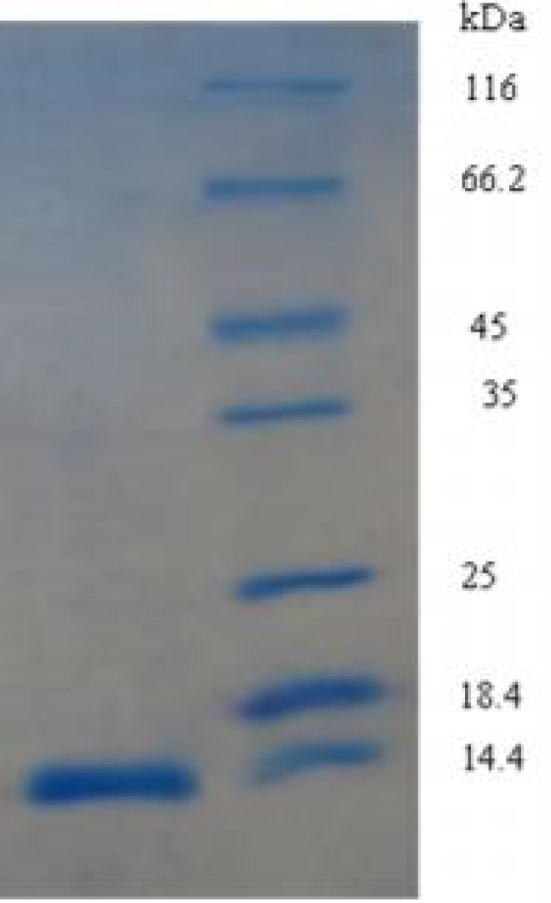
The over expressed recombinant HBsu protein. Left, HBsu protein; Right, Protein molecular size marker.

The first step in the study of the HU function in *H. karajensis* is the purification of this protein. There are reports about recombinant HU protein purification [24]. In our previous research we isolated the *hbs* gene from *B.subtilis* ATCC 6633 and the HBsu protein was expressed and purified (Fig. 1) [21], but this protein had an His tag and was differed from the native HBsu. In the current study, the wild type of HU was purified with a fast, two-step method.

**Figure 2:**
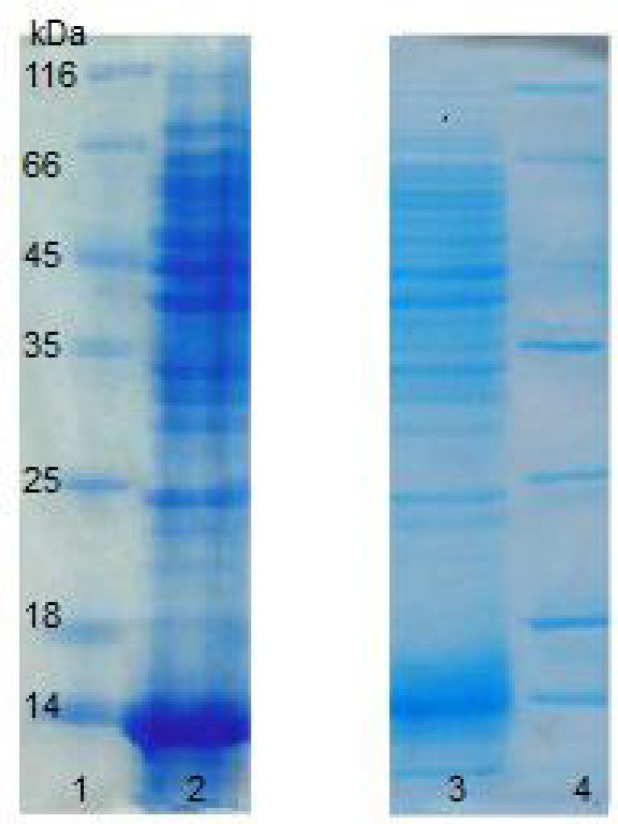
15% SDS PAGE ofprotein extraction, 120 V, Coomasssie Brilliant Blue staining. Lanes 1 and 4: Protein molecular size marker; Lane 2: Protein extraction of *Halobacillus karajensis*; Lane 3: Protein extraction of *Bacillus subtilis*

**Figure 3:**
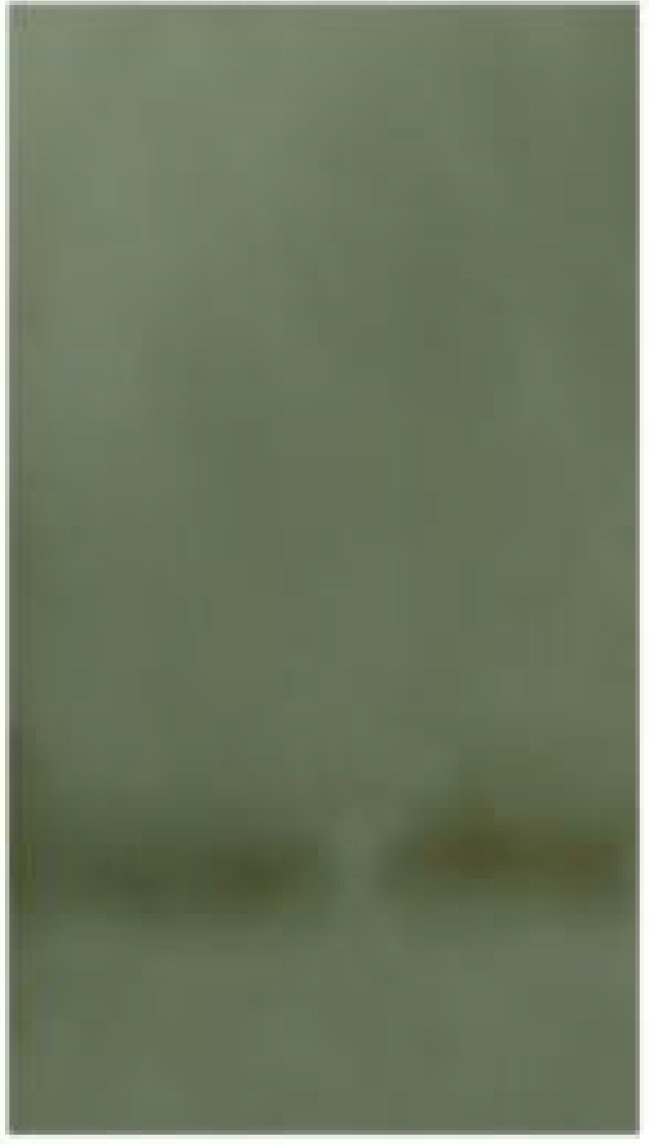
Immunoblotting with anti HBsu protein antiserum. Left, Protein extraction of *Halobacillus karajensis*; Right, Protein extraction of *Bacillus subtilis*

The protein extracted from *H.* *karajensis* and *B.subtilis* (as a control) was electrophoresed (Fig. 2) and immunoblotted with anti-HBsu antiserum [20], and a single band was observed (Fig. 3). This indicates that HU from *H.* *Karajensis* is immunologically similar to HU from *B.subtilis* (HBsu).

**Figure 4:**
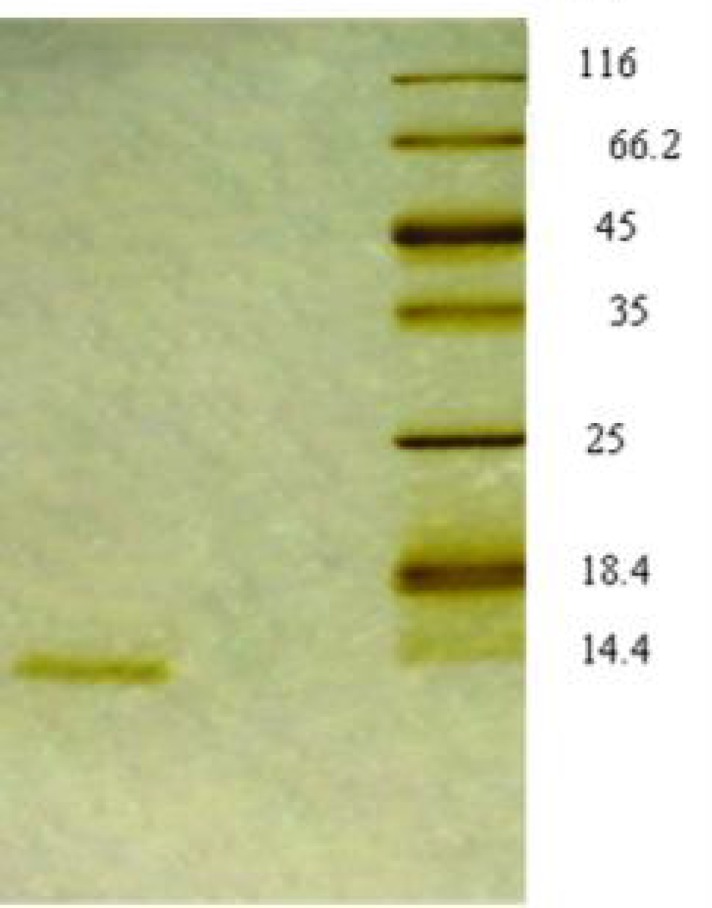
Silver stained 15% SDS poly acrylamide gel of chromatography fractions. Left, Fractions have not been adsorbed to the DEAE Sepharose CL-6B column; Right, Protein molecular size marker

**Figure 5:**
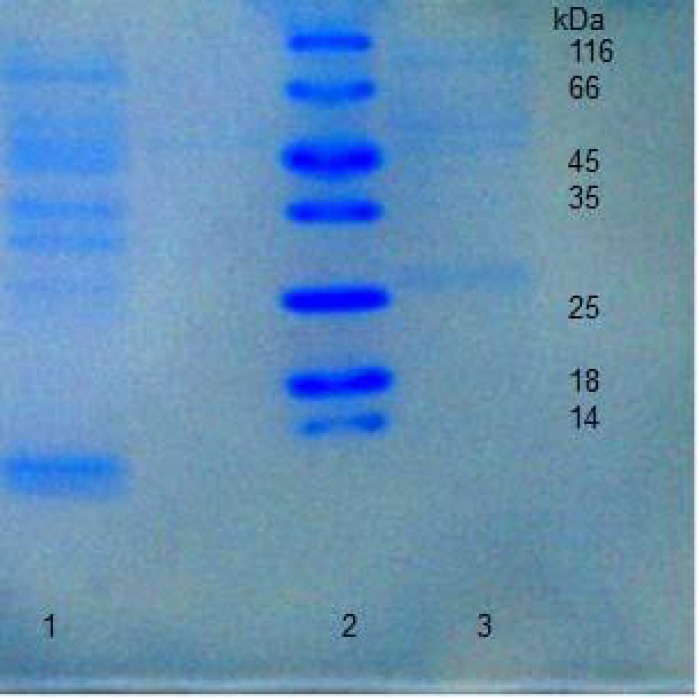
Coomasssie Brilliant Blue stained 15% SDS poly acrylamide gel of chromatography fractions eluted after 0.25-1 M NaCl gradient. Lane 1: 0.25 M NaCl; Lane 2: Protein molecular size marker; Lane 3: 0.5 M NaCl

In this research, the extracted protein from *H.* *karajensis* was precipitated with 45-90% ammonium sulfate solutions. The most appropriate sample containing a maximum amount of protein with a molecular weight similar to the HU protein belonged to the 65% precipitated sample. The protein was then loaded onto the anion exchange column DEAE-Sepharose CL-6B. The obtained fractions were loaded on 15% SDS poly acrylamide gel and stained with silver nitrate and Coomasssie Brilliant Blue (Figs. 4, 5). HU was not adsorbed to the column at this pH, hence a single band in primary fractions was observed. Since the HU protein is basic [3] and has a positive charge at pH 7.8 in buffer B, it cannot adsorb to the DEAE Sepharose CL-6B column at this pH. The other proteins, however, were not basic and were thus adsorbed to this column at this pH with different strengths, leading to the purification of the HU protein.

**Figure 6:**
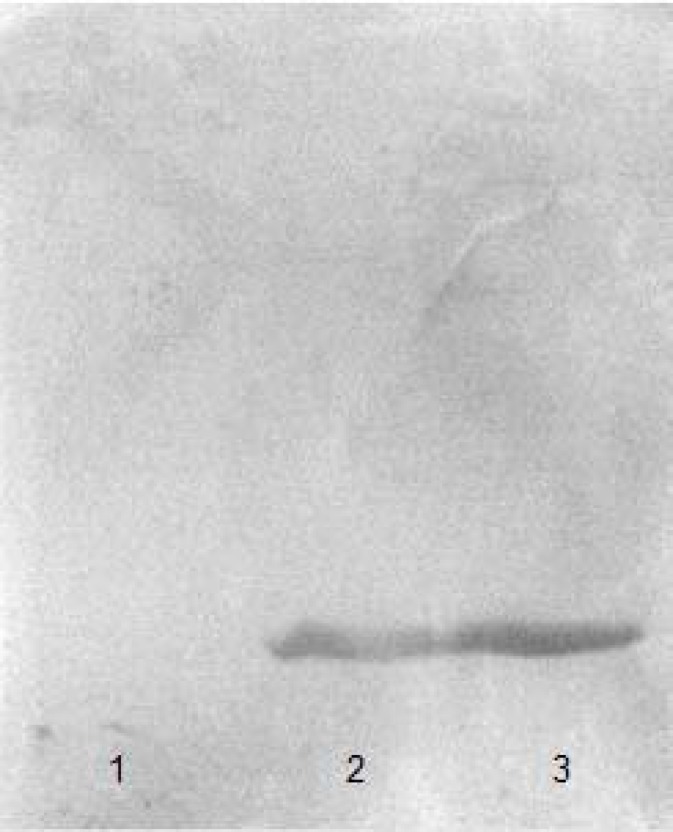
The proteins were transferred onto nitrocellulose membrane for Immunoblotting analysis performed using anti HBsu antiserum. Lane 1: Fractions were eluted with 0.25 M NaCl in buffer B; Lane 2: Fractions were not adsorbed to the column; Lane 3: HU protein extraction of *Bacillus subtilis*

The relative molecular weight of the single band in 15% SDS poly acrylamide gel was estimated to be about 11 kDa, similar to that of HBsu from *B. subtilis* [21]. For specific recognition of the HU protein, all fractions were immunoblotted with the produced monospecific polyclonal antiserum against HBsu (HU protein extraction of *Bacillus subtilis* being used as a positive control.) (Fig. 6), showing that the purified protein was HU.

In this study we used a fast and qualified method to purify a protein from *H. karajensis* which was immunologically similar to HBsu.
